# Prevalence of Hypertension and Diabetes and Coexistence of Chronic Kidney Disease and Cardiovascular Risk in the Population of the Republic of Moldova

**DOI:** 10.1155/2012/951734

**Published:** 2012-11-28

**Authors:** Igor Codreanu, Vera Sali, Sergiu Gaibu, Luminita Suveica, Sergiu Popa, Norberto Perico, Bogdan Ene-Iordache, Sergio Carminati, John Feehally, Giuseppe Remuzzi

**Affiliations:** ^1^Republican Clinic Hospital, Chisinau, Moldova; ^2^Municipal Council Health Department, Chisinau, Moldova; ^3^Clinical Research Center for Rare Diseases “Aldo e Cele Daccò” and Centro Anna Maria Astori, Science and Technology Park Kilometro Rosso, Mario Negri Institute for Pharmacological Research, 24126 Bergamo, Italy; ^4^The John Walls Renal Unit, Leicester General Hospital, Leicester LE5 4PW, UK

## Abstract

In 2005, the International Society of Nephrology (ISN) established the Global Outreach Program (GO) aimed at building a capacity for detecting and managing chronic kidney disease and its complications in low- and middle-income countries. Here we report data from the 2006-2007 screening program (1025 subjects from the general population) in the Republic of Moldova aimed to determine the prevalence of hypertension, diabetes, and their coexistence with microalbuminuria. The likelihood of a serious cardiovascular (CV) event was also estimated. Hypertension and diabetes were very common among screened subjects. The prevalence of microalbuminuria was 16.9% and that of estimated GFR <60 ml/min/1.73 m^2^ (decreased renal function) was 9.4%. Male gender was associated with an increased prevalence of hypertension and microalbuminuria. Hypertension and diabetes clustered in subjects with microalbuminuria and renal dysfunction. Risk factors such as preobesity/obesity, physical inactivity and smoking were relatively common, even in younger participants. The prevalence of subjects with predicted 10-year CV risk ≥10% was 10.0%. In conclusion, in the Republic of Moldova patients with hypertension and diabetes should be screened for the coexistence of renal abnormalities, with the intention of developing disease-specific health-care interventions with the primary goal to reduce CV morbidity and mortality and prevent renal disease progression to end stage renal disease.

## 1. Introduction

The growing global burden of noncommunicable chronic diseases (NCDs) worldwide has been disregarded until recently by policy makers, major aid donors, and academics. However, NCDs are the leading cause of death in the world [1–3]. In 2008, there were 57 million deaths globally of which 63% were due to NCDs. Cardiovascular disease (including hypertension), diabetes, cancer, and chronic respiratory disease are the four NCDs prioritized in the Global NCD Action Plan endorsed by the World Health Assembly in 2012 because they share major behavioral risk factors amenable to public-health action and are the major contributors to the global NCD burden. There is also evidence that chronic kidney disease (CKD) is a key determinant of the poor health outcomes of hypertension and diabetes and one of the strongest cardiovascular (CV) risk factors [4]. In the general population, glomerular filtration rate (GFR) lower than 60 mL/min/1.73 m^2^ and albuminuria—one of the earliest manifestation of CKD—are associated with an independent risk of CV morbidity and mortality [5–7]. Nevertheless, data on the prevalence of renal dysfunction and microalbuminuria from population screening programs are scarce, especially in low-income countries, where renal and CV risk factors, such as hypertension, diabetes, and obesity, are increasing at an even higher rate than in industrialized nations [8].

The Republic of Moldova remains the poorest country in Europe, ranking 111 according to the Global Human Development Index [9], with CV disease as the main cause of death [10, 11] and treatment options for patients with NCD are very limited, especially in rural areas. Renal replacement therapy (RRT) for end stage renal disease (ESRD) cannot be offered to all patients due to the shortage of resources. Since measurements of renal function and albuminuria are easy and relatively inexpensive, detection programs for kidney disease and its associated risk factors seem to offer a valuable opportunity to establish early prevention strategies, particularly at the primary-care level.

In 2005, the International Society of Nephrology (ISN) established the Global Outreach Program (GO) (formerly called the Commission on Global Advancement of Nephrology (COMGAN)) aimed at building global capacity for detecting and managing CKD in low- and middle-income nations (http://www.theisn.org/). In 2007, the ISN funded the establishment of an electronic database (Kidney Disease Data Center (KDDC)) to support the collection and analysis of data obtained through ISN-sponsored prevention programs. This program has been developed in the Republic of Moldova for general population screening, providing a unique opportunity to assess the prevalence of renal dysfunction and microalbuminuria in two representative urban areas of the country.

In the present study we analyzed data from the 2006-2007 screening program in Moldova with the aim of determining the prevalence of hypertension, diabetes, and their coexistence with renal dysfunction and microalbuminuria by using data from the ISN KDDC. Moreover, we estimated the likelihood of a serious CV event in the same population.

## 2. Methods

### 2.1. Study Population

This study is part of the screening and intervention program Early Detection and Intervention Program for Chronic Renal and Cardiovascular Disease in the Republic of Moldova on the behalf of ISN GO in two representative cities of the country. The screening was conducted in the general population invited to attend to two primary health care units in the capital Chisinau and in Ialoveni. During the period from September 2006 to January 2007, 1025 individuals aged 18 or more participated to the study (Chisinau: 553, Ialoveni: 472).

### 2.2. Data Collection

Data were collected prospectively using an electronic database provided to the Moldova Coordinating Center in Chisinau by the ISN KDDC based in Bergamo, Italy. Data quality was monitored by the bioinformatics team of the Clinical Research Center for Rare Diseases “Aldo e Cele Dacco,” Bergamo, Italy. In the day of screening, the participants were enrolled after being informed about the objectives of the survey and the screening procedure, as well as potential benefits of the study. Baseline data obtained from each participant included age, sex, marital and employment status, education level, smoking status, alcohol use, physical activity, diabetes mellitus status, and personal history of hypertension and kidney disease. Family history of diabetes, hypertension, cardiovascular diseases, and kidney disease was also collected. Height and weight were measured and used to calculate body mass index. Systolic and diastolic blood pressures were measured by trained personnel using manual sphygmomanometers after participants rested quietly for five minutes.

Fasting venous blood (creatinine, glucose, cholesterol, and hemoglobin) and spot urine (albumin, creatinine) specimens were provided by the participants according to local protocol. Individuals with microalbuminuria were asked to proceed with a second confirmatory test a week apart. Screening was carried out under research protocol approved in advance by the relevant institutional review board. All participants gave a written informed consent.

### 2.3. Definitions

Hypertension was classified according to the *Seventh Report of the Joint National Committee on Prevention, Detection, Evaluation, and Treatment of High Blood Pressure scheme* [12]. Participants were classified as having diabetes mellitus (DM) if they reported a history of or were currently treated for DM, or had a fasting blood glucose level ≥126 mg/dL. Participants' body mass index was classified according to the current World Health Organization (WHO) [13] scheme as follows: <18.49 kg/m^2^ (underweight), 18.5–24.9 kg/m^2^ (reference range), 25–29.9 kg/m^2^ (preobese), and ≥30 kg/m^2^ (obese). Microalbuminuria was defined as urinary albumin/creatinine (ACR) values of 30–300 mg/g. Subjects with ACR lower than 30 mg/g were defined as normoalbuminuric, and those with more than 300 mg/g as macroalbuminuric. Urine albumin concentration was determined by an ELISA method (UBI-MAGIWEL Enzyme Immunoassay) [14]. Serum creatinine was used to estimate glomerular filtration rate (GFR) using the 4-variable Modification of Diet in Renal Disease (MDRD) Study equation in all participants [15]. Because the MDRD Study equation is less accurate at higher levels of true GFR, participants were classified regarding the presence or absence of estimated GFR (eGFR) <60 mL/min/1.73 m^2^ (termed “decreased eGFR”).

The likelihood of a serious CV adverse outcome (death, myocardial infarction, stroke, heart failure, or coronary revascularization) during the next 10 years was estimated by using World Health Organization (WHO) charts. 

### 2.4. Analytical Methods

Descriptive statistics were tabulated using count and percentage or mean, as appropriate. Missing values were not imputed and casewise deletion did not occur; therefore the total number of participants varied by variable. Outcome of interest included hypertension, diabetes, body mass index, microalbuminuria, eGFR <60 mL/min/1.73 m^2^), and CV risk, as defined. According to the WHO charts, the 10-year risk of fatal or nonfatal CV event considered the following parameters: age (years), gender, smoking habit, SBP (mmHg), cholesterol (mg/dL), and presence/absence of DM. An SAS version 9.1 code was constructed to generate the 10-year CV risk class for each participant. Results were stratified by age, sex, and microalbuminuria. Statistical analyses were performed using Stata software, version 12.1 (StataCorp, http://www.stata.com/).

## 3. Results

### 3.1. Participant Characteristics

Demographic and clinical characteristics of the 1025 participants are listed in Table 1.

Mean age of participants was 48 years ranging from 18 to 77 years, and most of them were women (73.3%). Levels of education were variable, with the highest fraction of subjects with >10 years of education. Participants reported mainly working outside the home, but also a large rate of unemployment. Prevalence of current smoking habit was higher than 13%, with 35% of men and 5% of women being current smokers. Among current male smokers, microalbuminuria was detected in 17.6% of subjects and GFR <60 mL/min/1.73 m^2^ in 4.9%. Alcohol consumption was variable, but always higher in men than in women. Daily consumption of fruit and vegetables was common, while no more than 35% of participants reported daily inactivity with slight prevalence in women. Positive familiar history for hypertension and CV disease was relatively high. At physical examination mean BMI was in the preobese stage both in men and women, whereas mean blood pressure levels were within the normal range for both genders.

### 3.2. Prevalence of Hypertension, Diabetes and Preobesity

The prevalence of hypertension (assessed at the clinic visit) was high with more than 50% of participants having elevated SBP or DPB or were receiving treatment for previously diagnosed hypertension. As expected, the prevalence of hypertension was the highest in participants who were male, and older than 40 years (Table 2). Overall 62.7% of hypertensive subjects were taking antihypertensive medication. Hypertension was in general well controlled. 16.1% of participants had DM. No significant difference was found in the prevalence of DM between female and male subjects (Table 2). Seventy-eight percent of diabetic subjects had hypertension. The prevalence of DM progressively increased with BMI, and 5.6% of the preobese and 7.8% of the obese were diabetic. Thirty-seven percent of participants were preobese (assessed by BMI), with a prevalence lower in female than male.

### 3.3. Prevalence of Microalbuminuria and eGFR <60 mL/min/1.73 m^2^

The prevalence of microalbuminuria (assessed at the clinic visit) was 16.9%, higher in participants older than 40 years (Table 3). Microalbuminuria was more common in male than female. The prevalence of eGFR <60 mL/min/1.73 m^2^ was 8.2% (Table 3). Higher prevalence was found in participants who were male and older than 40 years. The proportion of patients who had both microalbuminuria or macroalbuminuria and eGFR <60 mL/min/1.73 m^2^ was 2.2%.

### 3.4. Microalbuminuria in Hypertension and Diabetes

We analyzed the relationship between microalbuminuria and hypertension or diabetes according to age groups and whether eGFR was lower than 60 mL/min/1.73 m^2^ (Table 4). In nondiabetic hypertensive subjects the prevalence of microalbuminuria was 19.0%. It was higher in participants with hypertension older than 60 years who had decreased eGFR (ACR 30–300 mg/g: 7.4%). Similarly, in diabetics the proportion of patients with microalbuminuria was 12.0%. In this cohort, the prevalence of microalbuminuria progressively increased with age independently of eGFR. Nevertheless, microalbuminuria was more frequent in patients with eGFR <60 mL/min/1.73 m^2^ (7.4%) than in those with higher eGFR (4.6%). Microalbuminuria was detected in 10.9% of subjects with coexistence of diabetes and hypertension (Table 4).

### 3.5. Distribution of Cardiovascular Risk

The prevalence of participants with predicted 10-year cardiovascular (CV) risk ≥10% was 10.0%. For predicted CV risk 10.1–20%, 20.1–30%, 30.1–40%, and >40.1%, the proportion of subjects was 5.35%, 3.33%, 0.81%, and 0.81%, respectively.

## 4. Discussion

The present screening program of a representative cohort of general population in Moldova showed a very high prevalence of hypertension. DM was also common among the screened participants, together with hypertension clustered in subjects with microalbuminuria, and renal dysfunction. In accordance with previously published studies, male gender was associated with an increased prevalence of hypertension, microalbuminuria and reduced renal function [16–18]. In addition to disease requiring medical treatment, risk factors such a pre-obesity/obesity, inactivity, and smoking were relatively prevalent in the population screened, even in younger participants. A large proportion of the participants were unemployed which would favor a sedentary lifestyle eventually contributing to the high prevalence of pre-obesity and ultimately of hypertension and DM.

Despite the prevalence of these medical and nonmedical risk factors, microalbuminuria was present in 10.9% of subjects with coexistent DM and hypertension. Raised blood pressure is a key NCD risk factor, and its prevalence, like that of diabetes, is projected to increase sharply over the next few decades, especially in low-income countries [19]. Therefore we speculate that—because the epidemic of NCD is still becoming established in the Republic of Moldova—the full impact of these risk factors has not yet been fully realized, and the prevalence of microalbuminuria and possibly CKD (defined as eGFR <60 mL/min/1.73 m^2^) are likely to increase substantially during the coming decades, in parallel with CV disease. This highlights the importance of prevention because most residents in this country cannot afford renal replacement therapies and coronary revascularization procedures.

The prevalence of microalbuminuria found in the present study is higher than that reported by other ISN-sponsored screening programs in indigent residents of Guadalajara, Mexico [20], in Nepal [21], and Kinshasa, Democratic Republic of Congo [22]. However this highlights the generalizability of this approach for case detection in diverse settings around the world. For example, the same approach has been adopted by the Prevention of Renal and Vascular ENd-stage Disease (PREVEND) study, a large European screening program [23].

About 10% of the subjects in the screened population had an estimated 10% or higher risk of developing a fatal or nonfatal CV event in the following 10 years. There is evidence that the increased cardiovascular risk in CKD patients does not just coexist with hypertension or DM. Indeed, an independent and progressive association between GFR and the risk of CV events and deaths has been found in a community-based study in more than 1 million adult subjects in the USA [24]. Similarly, a recent study in more than 6000 people followed on average 7 years has shown that the risk of cardiovascular death was increased 46% in subjects with a mild-to-moderate reduction in GFR, independent of conventional risk factors such as hypertension and diabetes [25]. Evidence is also available that the risk of mortality is better correlated with proteinuria/albuminuria than with GFR alone [6, 26–28]. A large population-based study of more than 1 million people from Alberta, Canada, demonstrated that the presence of proteinuria was associated with marked increase in the risk of all-cause mortality and the risk of kidney failure, independent of GFR and at all levels of baseline kidney function [29]. The association between proteinuria and CV mortality independent of hypertension, DM, and GFR has recently been demonstrated in a meta-analysis of 22 studies [7] including participants with a wide age range from around the world. The independent risk associated with albuminuria for all-cause mortality, CV mortality, and progression to ESRD was confirmed in over 1.1 million people with proteinuria identified only by detection of “trace” or greater on dipstick urinalysis, as well as in over 100,000 who had an albumin creatinine ratio (ACR) of 10 mg/g or more [7]. Thus, considering the high prevalence of microalbuminuria and renal dysfunction in the present screening in the Republic of Moldova, the predicted CV risk at 10 years was probably underestimated. Long term outcome analysis of the present screened cohort will clarify whether the inclusion of albuminuria and/or reduced GFR among the variables considered in algorithms for the prediction of individual CV risk will improve the performance of current WHO prediction charts.

Evidence is also emerging that CKD and CV diseases have a major impact on macroeconomic development due to diminished labor supply related to premature death and disability in people of working age. According to WHO, these conditions decrease the potential annual growth rate in gross domestic product by 1–5% in low-income countries experiencing a rapid economic growth [30]. Importantly, data from large trials have consistently shown that offpatent drugs such ACE inhibitors can reduce albuminuria and prevent GFR decline and CV events [31]. Thus, prevention program should identify subjects whose renal abnormalities can be treated early at low cost, with the primary goal to reduce CV mortality and morbidity, which, in turn, may translate into an economical benefit.

Screening the general population for CKD by measuring albuminuria and/or serum creatinine has been advocated to identify and treat those at risk for progressive renal disease, arguing that most persons with albuminuria and/or reduced GFR are asymptomatic; however, it is so far considered premature to recommend in both industrialized and low- and middle-income countries. On the other hand, screening in people at increased risk of CKD (so called “selective screening”) such as those with hypertension and/or DM, in whom early intervention can slow down the deterioration of renal function, has received more support [32]. Further studies, however, are warranted before definite recommendations for general population or selective screening for CKD can be provided. Difficulties in identifying subjects at increased risk of CKD in low-income countries should be considered. Therefore, in this setting, a prescreening phase including history, blood pressure, and anthropometric values might allow the identification of patients in whom screening with blood and urine testing could be most cost effective. There are also data from low-income countries that a significant percentage of people younger than 60 years without previous history of hypertension and diabetes has microalbuminuria/proteinuria [21]. Concerns about general population screenings include not only the cost of the screening itself but, more importantly, the risk and the cost of treating false positive subjects with no other modifiable risk factors. Thus, the first positive test should be always repeated and only those with confirmed positivity should be treated [33] as was done with the screening in the Republic of Moldova. When debating the issue of screening for NCDs, the perspective of the low- and middle-income countries should be considered as well as that of the industrialized nations. And, there is not a unique blueprint of screening strategy even among low-income countries, so that the approaches should be adapted to the conditions and socioeconomic status of each nation [34].

Our study has some limitations: the study cohort includes more than double women than men; results from this screening cannot be taken to infer the absolute or relative prevalence of hypertension, DM, and renal abnormalities in the Republic of Moldova, since all subjects were referred to two centers of the country. However, in consideration of the limited availability of local resources and relatively low population of the country, current data seem reasonably reliable which is supported by the fact that, inline with the available evidence, the prevalence of microalbuminuria was higher in subjects with hypertension or DM.

## 5. Conclusion

We found that hypertension, DM, microalbuminuria, and impaired kidney function are common within the general population of the Republic of Moldova. Thus patients with hypertension or DM should be screened for coexistence of renal abnormalities. Overall the present data demonstrate the feasibility of early detection of CKD and associated risk factors in low-income countries like Moldova. Top priority for the prevention of NCD including CKD must focus on effective methods to facilitate physical activity, control tobacco use, reduce harmful use of alcohol, and promote a healthy diet. In addition, individual disease-specific health-care interventions are also required to address those with hypertension, DM, CKD, or CV disease or at a high risk of developing these noncommunicable diseases.

## Figures and Tables

**Table 1:** Demographics and clinical characteristics of participants in the screening program.

	Overall (*n* = 1025)	Women (*n* = 737)	Men (*n* = 288)
Age (years)	48.8 ± 14.6	48.7 ± 14.5	50.0 ± 14.8
Education (years)	944	673	271
>10	343 (36.3)	231 (34.3)	112 (41.3)
6–10	234 (24.7)	166 (24.7)	68 (25.1)
1–5	312 (33.0)	232 (34.5)	80 (29.5)
None	55 (5.8)	44 (6.5)	11 (4.1)
Work	927	657	270
Physical labor	283 (30.5)	172 (26.2)	111 (41.1)
Office work	210 (22.6)	157 (23.9)	53 (19.6)
House work	83 (22.6)	72 (11.0)	11 (4.1)
Unemployed	351 (37.8)	256 (39.0)	95 (35.2)
Fruit/Vegetable intake	1018	732	286
1 x/day	545 (53.5)	420 (57.4)	125 (43.7)
3–5 x/day	355 (34.8)	227 (31.0)	128 (44.8)
1 x/week	101 (9.9)	73 (10.0)	28 (9.8)
None	17 (1.6)	12 (1.6)	5 (1.7)
Smoking	1021	734	287
Current	139 (13.6)	37 (5.0)	102 (35.5)
Former	33 (3.2)	5 (0.7)	28 (9.8)
None	849 (83.2)	692 (94.3)	157 (54.7)
Alcohol	1021	735	286
1 x/day	21 (2.1)	7 (1.0)	14 (4.9)
1 x/week	148 (14.5)	61 (8.3)	87 (30.4)
1 x/month	292 (28.6)	208 (28.3)	84 (29.4)
None	560 (54.8)	459 (62.4)	101 (35.3)
Physical activity (min/d)	1007	727	280
>60	372 (36.9)	269 (37.0)	103 (36.8)
30–60	134 (13.3)	82 (11.3)	52 (18.6)
<30	120 (11.9)	88 (12.1)	32 (11.4)
None	381 (37.8)	288 (39.6)	93 (33.2)
Family history			
CKD	209 (21.3)	171 (24.2)	38 (13.9)
HTN	498 (50.2)	373 (52.1)	125 (45.3)
DM	157 (16.1)	121 (17.2)	36 (13.1)
Patient's history			
CKD	384 (38.1)	313 (43.2)	71 (25.2)
HTN	480 (47.5)	341 (46.8)	139 (49.5)
DM	125 (12.5)	86 (12.0)	39 (13.9)
MI/CVD	253 (25.2)	186 (25.8)	67 (23.8)
Physical examination			
BMI (kg/m^2^)	1005	724	281
	27.72 ± 5.35	27.9 ± 6.0	27.4 ± 4.7
<18.5	61 (6.07%)	49 (6.8)	12 (4.3)
18.5–24.9	254 (25.27%)	177 (24.4)	78 (27.8)
25–29.9	369 (36.72%)	257 (35.5)	111 (39.5)
>30	321 (31.94%)	242 (33.4)	79 (28.1)
Blood pressure (mm Hg)			
SBP	130.2 ± 21.3	129.1 ± 22.2	133.1 ± 19.3
DBP	83.1 ± 11.3	82.5 ± 12.2	85.0 ± 10.3
Laboratory parameters			
A/C ratio (mg/g)	20.8 ± 22.1	20.3 ± 19.8	22.1 ± 26.9
eGFR (mL/min/1.73 m^2^)	84.2 ± 21.3	79.1 ± 16.8	97.9 ± 25.6

Data are a number of screened subjects and percent (%) if not otherwise specified. Values for Age, BMI, SBP, DBP, A/C ratio, and eGFR are mean ± SD. CKD: chronic kidney disease; HTN: hypertension; DM: diabetes mellitus; MI/CVD: myocardial infarction/cardiovascular disease; BMI: body mass index; SBP: systolic blood pressure; DBP: diastolic blood pressure; A/C: urinary albumin/creatinine ratio; eGFR: estimated glomerular filtration rate.

**Table 2:** Prevalence of hypertension and diabetes in the Moldova population aged ≥18 years.

	Subjects (*n*)	Hypertension*	Subjects (*n*)	Diabetes°
All	991	520 (52.4)	959	155 (16.1)
Age (years)				
18–39		41 (7.9)		14 (9.0)
40–59		253 (48.8)		69 (44.5)
≥60		224 (43.2)		72 (45.4)

Women	716	369 (51.5)	695	105 (15.1)
Age (years)				
18–39		28 (7.6)		8 (7.6)
40–59		182 (49.4)		45 (42.8)
≥60		158 (42.9)		52 (49.5)

Men	275	151 (54.9)	264	50 (18.9)
Age (years)				
18–39		13 (8.6)		6 (12.0)
40–59		71 (47.3)		24 (48.0)
≥60		66 (44.0)		20 (40.0)

Data are a number of screened subjects and percent (%) if not otherwise specified. *Hypertension is defined as participants with SBP >140 mm Hg or DBP >90 mm Hg, or receiving treatment for previously diagnosed hypertension. °Diabetes is defined as participants reporting a history of, or currently treated for diabetes mellitus, or with fasting blood glucose level ≥126 mg/dL.

**Table 3:** Prevalence of microalbuminuria and decreased GFR.

	Subjects	ACR*	Subjects	eGFR°
	(*n*)	30–300 mg/g	(*n*)	<60 mL/min/1.73 m^2^
All	978	166 (16.9)	973	92 (9.4)
Age (years)				
18–39		43 (25.9)		5 (2.0)
40–59		68 (40.9)		28 (6.1)
≥60		54 (32.5)		58 (21.9)

Women	702	108 (15.3)	708	78 (11.0)
Age (years)				
18–39		31 (28.7)		4 (5.1)
40–59		41 (37.9)		24 (30.7)
≥60		36 (33.3)		50 (64.1)

Men	276	59 (21.3)	265	14 (5.2)
Age (years)				
18–39		12 (20.3)		1 (7.1)
40–59		27 (45.7)		4 (28.5)
≥60		18 (30.5)		9 (64.2)

Data are a number of screened subjects and percent (%) if not otherwise specified. ACR: urinary albumin/creatinine ratio. eGFR: estimated glomerular filtration rate. Microalbuminuria: ACR 30–300 mg/g; decreased GFR: eGFR <60 mL/min/1.73 m^2^.

**Table 4:** Prevalence of microalbuminuria in participants with hypertension or DM according to age groups and level of renal function.

	ACR (30–300 mg/g)*			
Age (years)	18–39	40–59	≥60	All ages
Hypertension				
eGFR (mL/min/1.73 m^2^)				
<60	1 (1.1)	4 (4.3)	7 (7.4)	12 (12.8)
≥60	8 (0.9)	25 (2.9)	20 (2.3)	53 (6.2)
Diabetes				
eGFR (mL/min/1.73 m^2^)				
<60	0 (0.0)	3 (3.2)	4 (4.3)	7 (7.4)
≥60	8 (0.9)	14 (1.6)	17 (2.0)	39 (4.6)
Diabetes + hypertension				
eGFR (mL/min/1.73 m^2^)				
<60	0 (0.0)	3 (3.3)	4 (4.3)	7 (7.6)
≥60	2 (0.2)	12 (1.4)	15 (1.7)	29 (3.3)

Data are a number of screened subjects and percent (%) if not otherwise specified. ACR: urinary albumin/creatinine ratio. eGFR: estimated glomerular filtration rate. Microalbuminuria: ACR 30–300 mg/dL. *Hypertension is defined as participants with SBP >140 mm Hg or DBP >90 mm Hg, or receiving treatment for previously diagnosed hypertension. °Diabetes is defined as participants reporting a history of, or currently treated for diabetes mellitus, or with fasting blood glucose level ≥126 mg/dL.
